# Micro-CT study of the dehiscences of the tympanic segment of the facial canal

**DOI:** 10.1007/s00276-016-1744-4

**Published:** 2016-09-16

**Authors:** Magdalena Kozerska, Janusz Skrzat, Alexandru Spulber, Jerzy Walocha, Sebastian Wroński, Jacek Tarasiuk

**Affiliations:** 1 Department of Anatomy, Jagiellonian University Medical College, ul. Kopernika 12, 31-034 Kraków, Poland; 2 Faculty of Physics and Applied Computer Science, AGH University of Science and Technology, al. Mickiewicza 30, 30-065 Kraków, Poland

**Keywords:** Facial canal, Temporal bone, Computed microtomography

## Abstract

**Purpose:**

To depict the anatomy of the tympanic segment of the facial canal using a 3D visualization technique, to detect dehiscences, and to evaluate their frequency, location, shape, and size.

**Methods:**

Research included 36 human temporal bones (18 infant and 18 adult samples) which were scanned using a Nanotom 180N device. The final resolution of the reconstructed object was 18 µm. Obtained micro-CT data were subsequently processed by the volume rendering software.

**Results:**

The micro-CT study allowed for the 3D visualization of the tympanic segment of the facial canal and detects dehiscences in the studied material in both infants and adults. Most of the dehiscences (66.7 %) involved the inferior wall of the tympanic segment in infants as well as in adults, and were located above and backward to the oval window. The most frequent dehiscence shape was elliptic (66.7 % in infants; 50 % in adults). Furthermore, we observed dehiscences of fusiform and trapezoidal shape in infants. Length of the dehiscences in most cases ranged from 0.5 to 1.4 mm (50 % in infants; 75 % in adults).

**Conclusions:**

Volumetric reconstructions demonstrated the course of the tympanic segment of the facial canal and its relationship with the tympanic cavity. Knowledge about the size and location of any dehiscence within the tympanic segment of the facial canal is necessary due to the surgical significance of this region. If a dehiscence occurs, there is an increased risk of injury to the facial nerve during the operations or spread of inflammation from the middle ear.

## Introduction

The facial canal (Fallopian canal) begins at the point where the facial nerve enters the petrous bone through the fundus of the internal acoustic canal and terminates in the stylomastoid foramen, which is situated between the styloid and mastoid processes of the temporal bone. There are three segments of the facial canal: the labyrinthine, tympanic, and mastoid segments. During its trajectory through the petrous bone, it undergoes two turns and gives apertures for the passage of the greater superficial petrosal nerve, stapedial nerve, and chorda tympani [9, 11, 22].

The tympanic segment of the facial canal lies between the labyrinth and the tympanic cavity. The anterior part of this segment runs from the geniculate ganglion (first genu), medially and above to the cochleariform process. Then, it runs along and within the medial wall of the tympanic cavity, parallel and below to the lateral semicircular canal, and above the oval window. The posterior part of the tympanic segment lies in the vicinity of the pyramidal eminence and then passes into the second genu. The length of the tympanic segment of the facial canal varies from 9 to 12 mm (mean length of 10 mm) and its width ranges from 1.2 to 1.6 mm [11, 12, 24].

The tympanic segment of the facial canal may have dehiscences of the wall which some researchers treat as an anatomical variant, whereas others regard this feature as a pathological condition. Although incidence of dehiscences is most common in the tympanic segment, it can also be found at the level of the geniculate ganglion or in the mastoid segment of the facial canal. A dehiscence is a sort of deficiency within part of the bony wall of the facial canal, with clearly marked edges. Hence, it should not be confused with a lack of bony wall caused by poor mineralization. The diameter of dehiscences of the facial canal varies from 0.4 to 2.8 mm [11]. In turn, a diameter smaller than 0.4 mm indicates rather on aperture for blood vessels [1].

The risk of facial nerve injuries increases in the case of occurrence of dehiscences of the facial canal wall. This wall can be locally incomplete, exposing the facial nerve to the tympanic cavity [12]. Potential inflammation may also spread through such dehiscences and involve the facial nerve. The incidence of dehiscence is correlated with the occurrence of facial palsy, chronic otitis media, cholesteatoma, longstanding inflammation, and tumors [2, 6–8, 14].

The presence of dehiscence is closely related to the ossification process of the facial canal. Two centers of ossification fuse in newborns in the tympanic segment near the oval window [18]. Therefore, the occurrence of dehiscences in this region can result from varied patterns of the ossification process. In turn, Yetiser reported that the fibrous layer which surrounds the facial nerve may have influence on the final architecture of the facial canal [25]. The micro-CT study performed by Skadorwa et al. revealed that the facial canal was visible in fetuses over 18 weeks of gestation; however, full ossification was as yet unfinished in the week 27 of gestation. Inability to visualize the anterior wall of the facial canal in fetuses below 27 weeks due to the later ossification of this part of the facial canal may explain why bony dehiscences occur more often in the tympanic segment [17]. In addition, Spector noticed that dehiscences of the facial canal can be caused by gestational aberrations in weeks 21–26, involving inability of the ossification centers to fuse [18].

The subject of our research was morphometrical analysis of the tympanic segment of the facial canal using microtomography, due to the high clinical significance of this segment. In this region, the facial nerve is regarded as the most vulnerable structure to injuries which may occur during surgical management.

Up until now, high-resolution images and accurate-volume reconstructions of the facial canal dehiscences have not been presented in the literature. This study was aimed at performing detailed three-dimensional reconstruction of the tympanic segment of the facial canal and imaging potential dehiscences within its wall using micro-CT data. Hereby, we estimated the frequency of observed dehiscences in infant and adult samples and depicted their morphological pattern and location. Presented micro-CT data of the facial canal dehiscences can give a better insight into their morphological variation and dictate new solutions for how to avoid potential damage of the facial nerve during surgical treatment within the tympanic cavity.

## Materials and methods

Micro-CT study of the tympanic segment of the facial canal was performed on 36 dry human temporal bones: 18 infant samples aged from 40 weeks of gestation to 7 years (7 paired bones and 4 unpaired: 3 left and 1 right bone) and 18 adult samples (9 paired bones) aged approximately from 18 to 35 years (10 female and 8 male samples). Age and sex of individuals were determined by the conventional anthropological methods. The temporal bones were harvested from skulls exposed during archaeological excavations conducted in the southern part of Poland. The skeletal material was dated as being from the fifteenth to seventeenth centuries. All examined samples were well preserved and visual examination did not reveal any damages or pathologic malformations. The temporal bones were scanned using a Nanotom 180N device produced by GE Sensing & Inspection Technologies Phoenix X-ray Gmbh. The micro-CT system provides unique spatial and contrast resolution of the scanned samples, because of the installed ultra-high performance nanofocus X-ray tube (180 kV/57 W) and tungsten target with diamond window. The working parameters of X-ray tube were *I* = 250 µA and *V* = 70 kV. The reconstruction of scanned samples was done with the aid of proprietary GE software datosX ver. 2.1.0 using the Feldkamp algorithm for cone beam X-ray CT [3]. The tomograms were registered on a Hamamatsu 2300 × 2300 pixel detector. The final resolution of the reconstructed objects was 18 µm. The post-reconstruction data treatment (denoising, cropping, and 16 bit to 8 bit conversion) was performed by means of the VGStudio Max 2.1 software (http://www.volumegraphics.com/en/products/vgstudio-max/). Further, series of micro-CT slices were converted into volumetric (3D image) data and processed with the CTVox volume rendering software supplied by the SkyScan company (http://bruker-microct.com/products/downloads.htm). This software enables 3D visualization of volumetric data using volume rendering techniques and interactively explores 3D voxel data by adjusting their transparency and color. Appropriate setting of clipping planes allowed the accurate visualization of rendered structures of the tympanic cavity, course of the facial canal, and any dehiscences within its wall. Using volume rendered images of the dehiscences, we measured their length on images subjected to the ImageJ Software—an open platform for scientific image analysis (https://imagej.nih.gov/ij/).

We quantified arbitrarily the size of the dehiscences into three categories termed as: small, medium, and large. The numerical interval of each category was established according to the range of variation of the length of dehiscences (0.5–3.5 mm) divided into three equal bins. Therefore, we obtained following categories: 0.5–1.4, 1.5–2.4, and 2.5–3.5 mm. In this way, we wanted to find out what is the most frequent size of dehiscences in the studied material, if it depends on sex and age of individual. Frequency of the dehiscences of the facial canal in each category was presented in the bar chart.

## Results

**Fig. 1:**
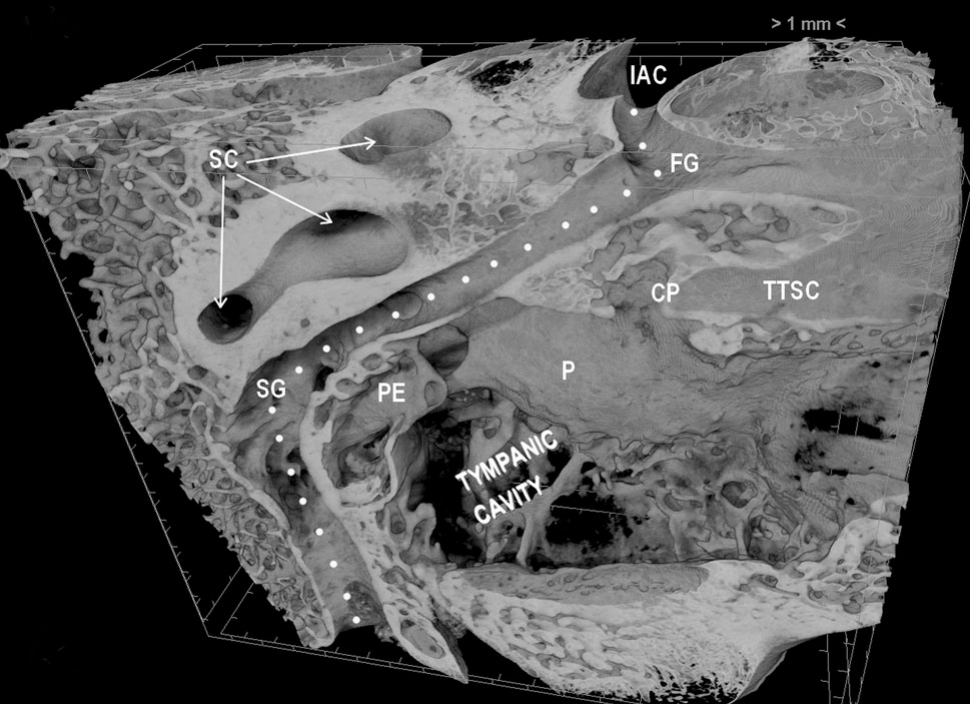
Representative volume reconstruction of the right facial canal and neighboring osseous structures of the middle and inner ear presented in the supero-lateral view. *FG* first genu, *SG* second genu, *IAC* internal acoustic canal, *SC* semicircular canals, *P* promontory, *CP* cochleariform process, *TTSC* semicanal for the tensor tympani muscle, *PE* pyramidal eminence

A volume rendered image of the facial canal and its relation to the neighboring structures is presented in Fig. 1. This 3D reconstruction shows subsequent parts of the facial canal: labyrinthine, tympanic, mastoid, and both genua (first and second genu). All these structures were well visible in volume reconstructions performed on infant and adult samples.

**Fig. 2:**
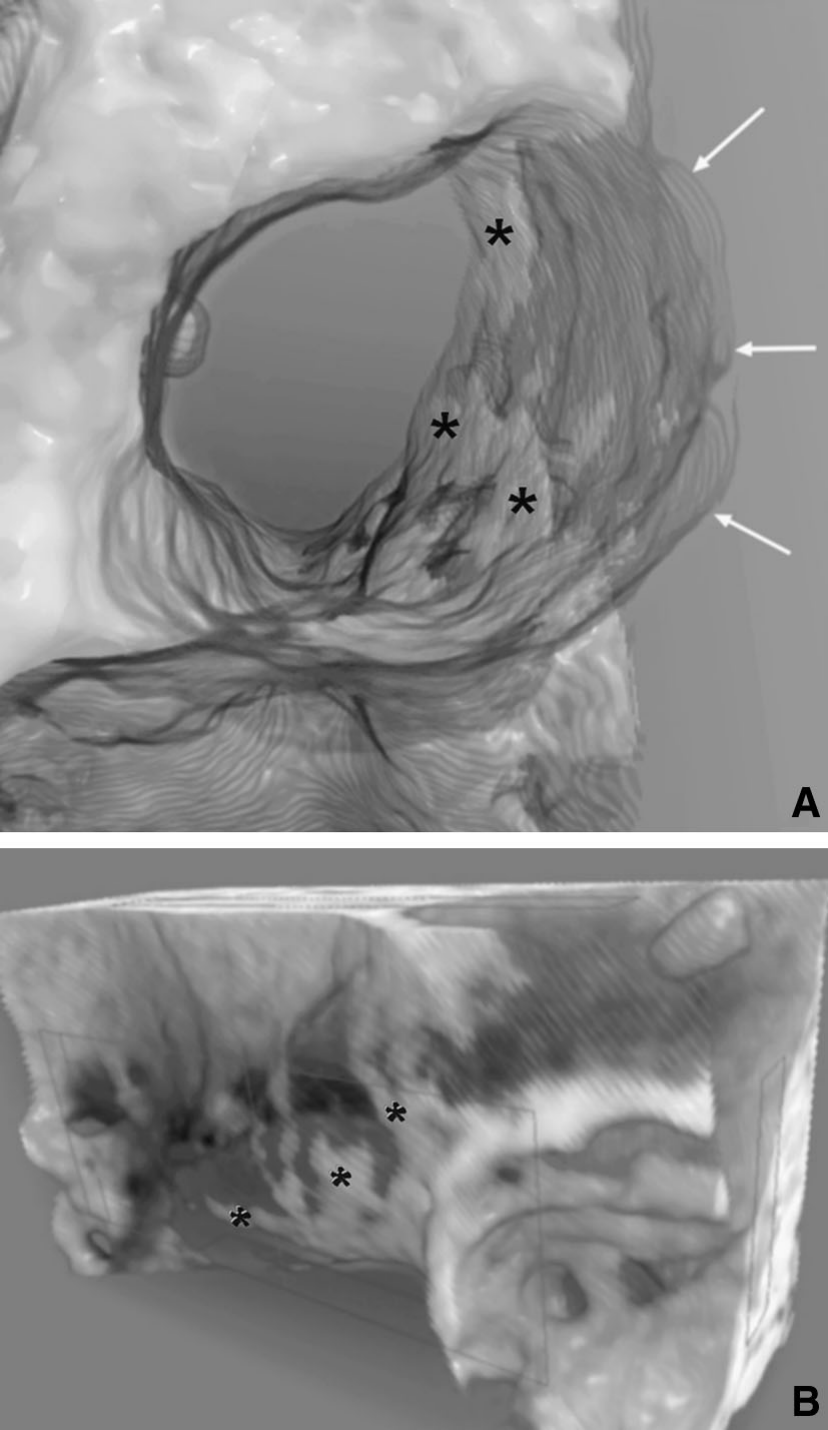
Transverse section of the facial canal (**a**) and lateral view on the lateral wall of the tympanic segment of the facial canal (**b**) presented as a volume rendering of the micro-CT data of the right temporal bone. Note that the lateral wall partially appears as a semitransparent lamina (indicated by the *arrows*), and shows local, well-mineralized spots (marked by *asterisks*)

Volume rendering of the micro-CT data revealed the potential defects of the facial canal’s wall, which could be caused by pathological factors or disturbances in the development of the facial canal. Obtained 3D reconstructions delivered visual information which was used to differentiate between typical dehiscences and osseous discontinuities resulting from poor mineralization of the wall of the facial canal (Fig. 2). Such a partial lack of the osseous wall within the tympanic segment was observed in 4 of all 18 adult samples (22.2 %). In the infant samples, integrity of the osseous wall of the tympanic segment of the facial canal was preserved, except in the cases with obvious dehiscences whose locations, sizes, and shapes were presented in Table 1, and compared with similar defects observed in adult individuals.

**Table 1:** Evaluation of the location, shape, and length of the dehiscences within the tympanic segment of the facial canal in infant and adult samples

SN	Sex	Left temporal bone			Right temporal bone		
		Location	Shape	Length (mm)	Location	Shape	Length (mm)
Infants							
24	F	Infero-lateral	E	3.02	Infero-lateral	E	3.47
29	F	Infero-medial	E	1.90	Infero-medial	E	1.88
45	M	Inferior	E	0.54	Inferior	F	1.03
6	NE	Inferior	E	1.06	Inferior	E	2.00
55 II	M	Inferior	F	1.23	Inferior	T	1.40
35	NE	Inferior	F	3.11	UL	UL	UL
43 II	M	DNP	–	–	Inferior	E	1.25
43 I	M	DNP	–	–	DNP	–	–
2.11	NE	DNP	–	–	UL	UL	UL
27	NE	DNP	–	–	UL	UL	UL
23	NE	UL	UL	UL	DNP	–	–
Adults							
11	F	Inferior	E	1.32	Infero-medial	E	2.84
40	F	Inferior	E	0.91	Inferior	R	0.65
44 III	F	Infero-medial	R	0.68	Inferior	R	0.57
64	F	Infero-medial	R	0.78	Infero-medial	R	1.36
70	F	DNP	–	–	Inferior	E	3.13
7 II	M	Inferior	R	1.17	Inferior	E	1.62
1	M	Inferior	E	1.02	DNP	–	–
7 III	M	DNP	–	–	DNP	–	–
12	M	DNP	–	–	DNP	–	–

*SN* sample number, *F* female, *M* male, *NE* not estimated sex, *DNP* dehiscence not present, *E* elliptic, *R* round, *F* fusiform, *T* trapezoidal, *UL* unilateral lack of the temporal bone

**Fig. 3:**
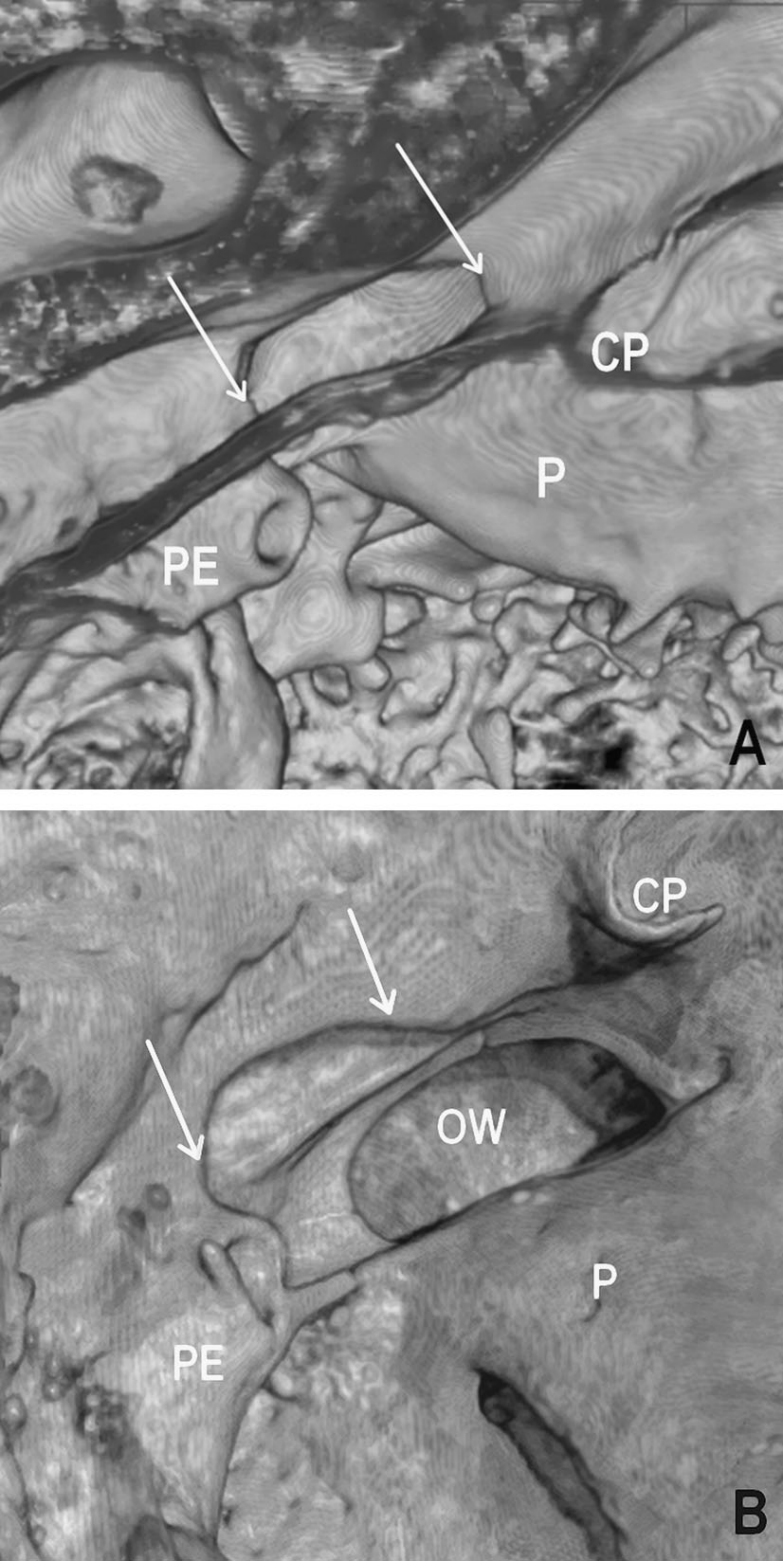
Volume reconstructions showing infero-medial (**a**) and infero-lateral (**b**) locations of the dehiscence within the wall of the tympanic segment of the right facial canal; *P* promontory, *CP* cochleariform process, *PE* pyramidal eminence, *OW* oval window, dehiscence of the tympanic segment marked by the *arrows*

Figure 2 presents uneven bone mineral distribution in the lateral wall of the tympanic segment of the facial canal. Therefore, in such a case, parts of the facial nerve can be unprotected by the bone and these bony defects may mimic dehiscences of the facial canal. We regarded this as a type of hollow facial canal with local tiny gaps of irregular outline which are irregularly scattered, whereas a typical dehiscence is restricted to a certain portion of the facial canal and has a defined contour (Fig. 3).

Observed frequency of dehiscences in the studied temporal bones was equal in infants and adults: 66.7 % (12 dehiscent tympanic segments per 18 examined infant samples and 12 dehiscent tympanic segments per 18 adult samples). Bilateral dehiscences of the tympanic segment were present in most of the individuals: infant samples 83.3 %; adult samples 71.4 %, whereas unilateral dehiscences occurred in infants in 16.7 % of cases and in adults in 28.6 %.

### Location and morphometry of the dehiscences

**Fig. 4:**
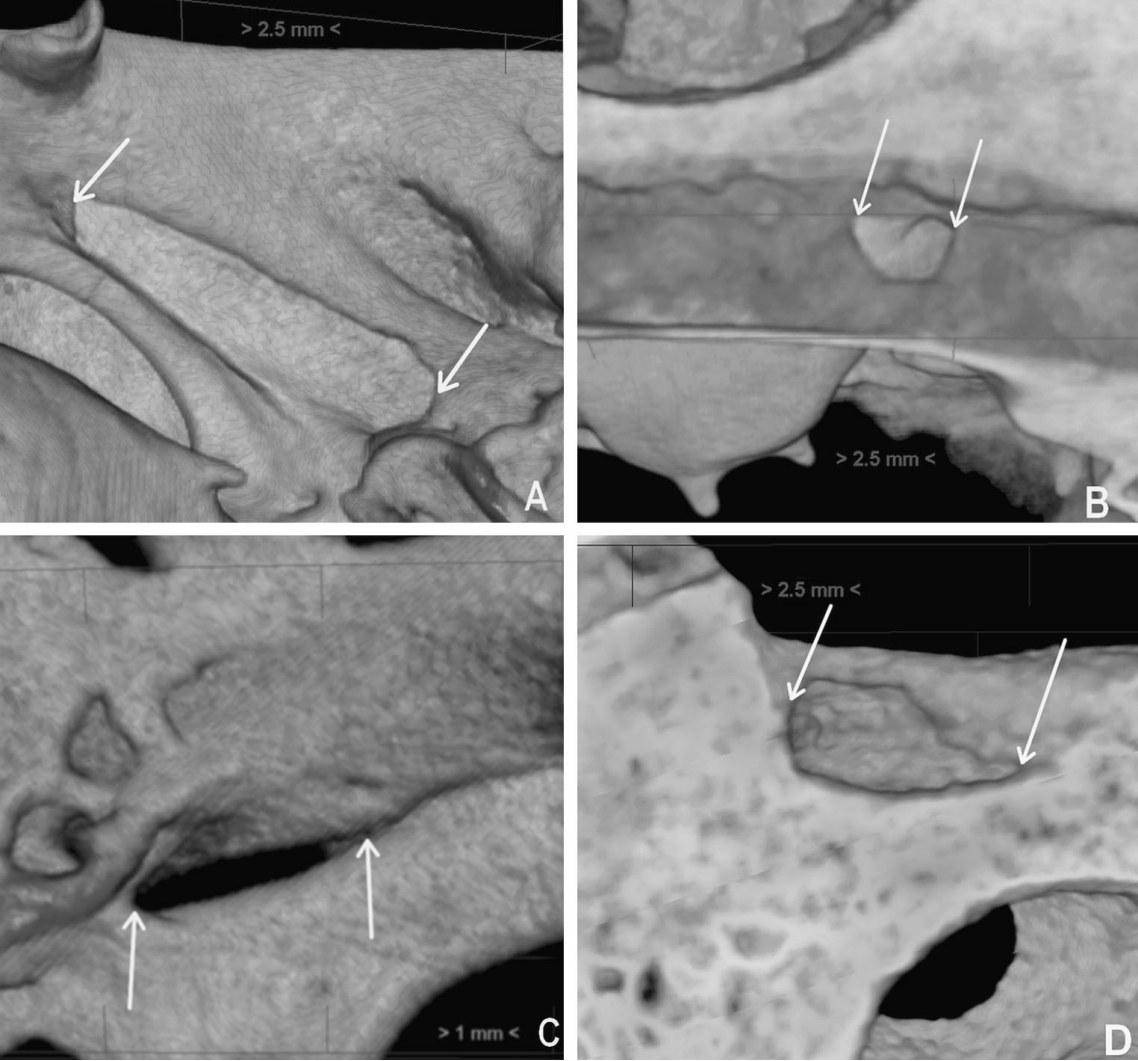
Shapes of the dehiscences of the tympanic segment of the facial canal (indicated by *arrows*) presented in close-up volume rendering: elliptic (**a**), round (**b**), fusiform (**c**), and trapezoidal (**d**)

**Fig. 5:**
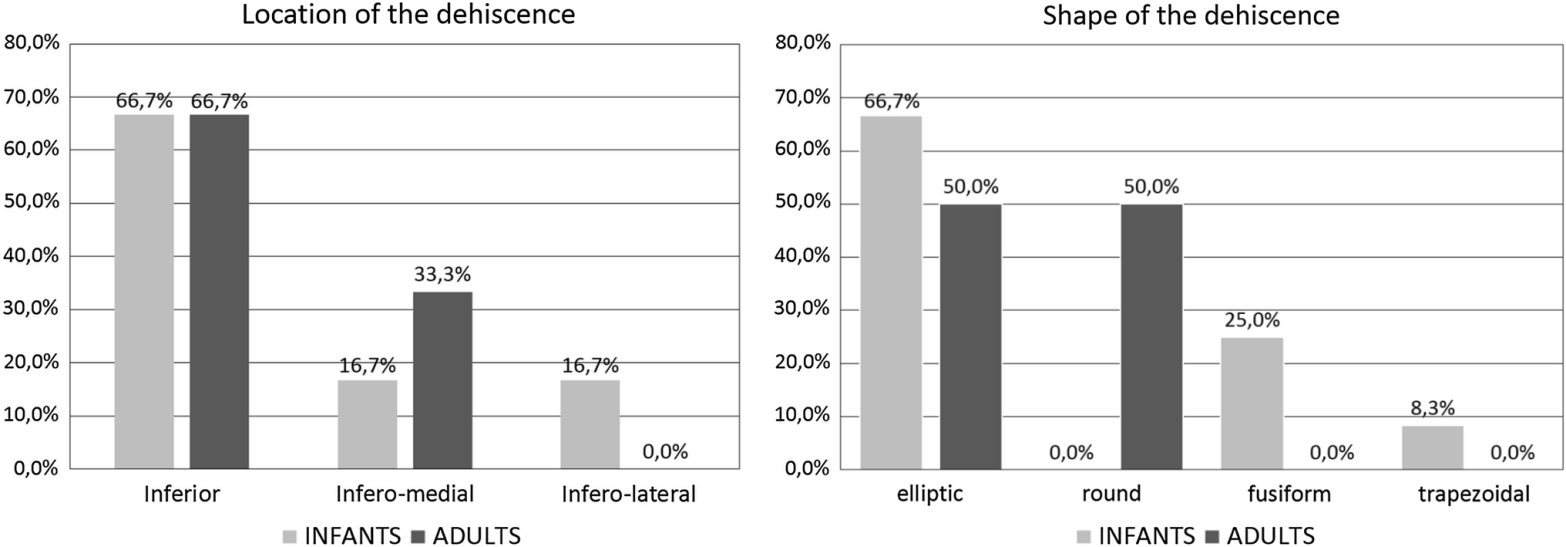
Percentage comparison of the locations and shapes of the dehiscences of the tympanic segment in age categories

Dehiscences of the tympanic segment were observed over the oval window or slightly backward of it, towards the second genu of the facial canal (Fig. 3). We did not find any dehiscences within the superior portion of the tympanic segment. Morphological appearance of the different types of dehiscences found in the wall of the tympanic segment of the facial canal was presented in Fig. 4. In examined material dehiscences occurred within the following portions of the tympanic segment of the facial canal: inferior, infero-medial, and infero-lateral. Their frequencies are presented in the bar chart in Fig. 5.

Both in the infant and adult samples, most of the dehiscences (≥50 %) were of elliptic shape; however, in infants, we also found dehiscences of fusiform and trapezoidal shapes. In turn, in infants, we did not observe round shaped dehiscences which occurred often in adults (50 %). The frequencies of distinct patterns of the shapes of the dehiscences and their locations in infant and adult samples are presented in Fig. 5.

**Fig. 6:**
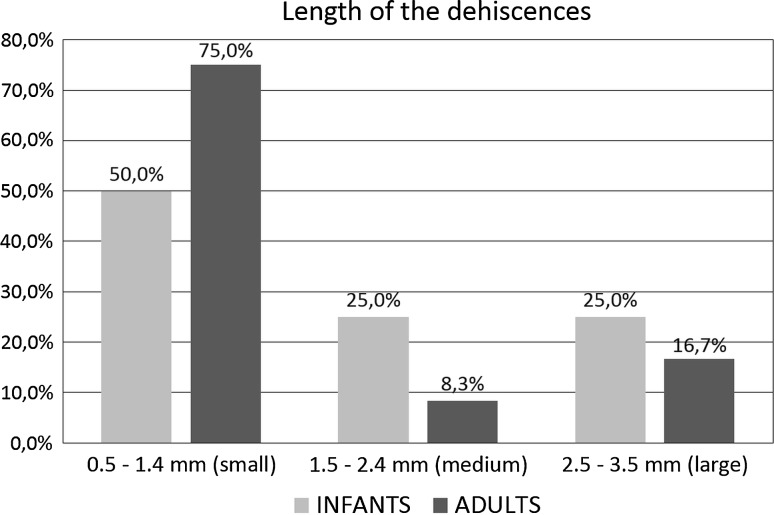
Percentage comparison of the lengths of dehiscences of the tympanic segment in age categories

The length of measured dehiscences of the tympanic segment of the facial canal ranged from 0.5 to 3.5 mm in the entire studied material. In the majority of cases, the dehiscences were of small size (0.5–1.4 mm) both for infant (50 %) and adult (75 %) samples. Longer dehiscences (medium size 1.5–2.4 mm, and large size 2.5–3.5 mm) were more frequent in infants (50 %) than in adults (25 %) (Fig. 6). Usually, the lengths of the bilateral dehiscences were similar between the right and left sides of individuals with the dehiscences in the tympanic segment. Bilateral dehiscences in the tympanic segment of the facial canal were observed in the same length interval in 80 % of the infant individuals and in 60 % of the adult individuals.

## Discussion

Up until now, the facial canal was the subject of morphological studies based on clinical computed tomography and histological sectioning followed by microscopic observation. The ability of clinical computed tomography to characterize microstructure of the facial canal, particularly in a three-dimensional manner, is considerably restricted. This is mainly caused by the tortuous course and relatively small transverse diameter of the facial canal (approximately 3.0 mm or less, depending on the segment of the facial canal). Usually, clinical CT scanners deliver images with resolution in the millimeter range which is not sufficient for performing accurate three-dimensional reconstructions of the facial canal. Although the Accuitomo 170 3D cone beam computed tomography has a fine enough resolution to identify the dehiscence in the clinical settings (voxel size: 0.08 × 0.08 × 0.08 mm, valid resolution: 0.1 mm), the 3D reconstructions of the facial canal will not be as good as those created from micro-CT scans. Contemporary micro-CT scanners can achieve resolution on the order of 1 μm. Therefore, we applied microtomography to obtain reconstructed objects with a resolution of 18 µm, which appeared accurate enough to visualize the facial canal morphology and its course in a three-dimensional manner. This imaging modality was used in only a few studies to present the overall course and morphology of the facial canal but not its structural defects [16, 17]. Although micro-CT is excellent for visualizing morphological aspects of the osseous defects of the facial canal, computed tomography still remains the fundamental technique for imaging facial canal anatomy in preoperative practice [8]. According to Fuse et al., the computed tomography results coincide with surgical findings in 75 % of cases with 66 % sensitivity and 84 % specificity [4]. Yetiser noted that computed tomography has some limitations in the evaluation of the dehiscences of the facial canal. In his opinion, minor defects of the facial canal can remain undetected by CT scanning, because the canal has a multiplanar and tortuous route [25].

Application of the computed microtomography allowed distinguishing typical dehiscences from apparent discontinuities of the osseous wall of the facial canal. Lacks of the osseous tissue in the wall of the facial canal captured by the micro-CT scanner were probably caused by weak or uneven levels of mineralization. If such samples were evaluated by clinical tomography, they might have been erroneously treated as dehiscences or undetected, because of their small size.

Thanks to imaging of the tympanic segment of the facial canal with pixel size of 18 µm, we could eliminate seeming dehiscences and estimate the incidence of real dehiscences. In this way, we obtained result indicating that the percentage of dehiscences was equal in infants and adults (66.7 %) which were not afflicted with any ear disease.

In literature, there are data which indicate an increasing risk of facial canal dehiscences on the presence of certain diseases. Genc et al. found the coexistence of scutum defect and facial canal dehiscence in 55.6 % of examined cases, suggesting that more attention should be paid to patients who have scutum defect to avoid facial nerve injury during middle ear operation [5].

Ocak et al. evaluated the prevalence of facial canal dehiscence in patients who underwent tympanoplasty. They found out that the prevalence of dehiscence was significantly higher in the patients with cholesteatoma (26 %) and negatively affected tympanoplasty outcomes, including hearing results, and the need for revision surgery [14]. Gülüstan et al. performed a study that showed increased incidence of dehiscences of the facial canal in patients with cholesteatoma (23.6 %) which affected mostly the tympanic segment (83.5 %). They also found that the facial canal dehiscence was 24.2-fold more common in patients with, lateral semicircular canal fistula and 4.1-fold more common in patients with destruction in the posterior wall of the external auditory canal [6]. Similar occurrence (27.1 %) of the facial canal dehiscences in patients with cholesteatoma was reported by Magliulo et al. who also found that in the majority of cases, they involved the tympanic segment [8].

Contrary, the otosclerosis incidence of facial canal dehiscences is significantly lower than in normal temporal bones (49.6 versus 65.7 %; *p* = 0.019) or occur sporadically (3.27 % of patients who had a stapedotomy). This was explained by the ability of the otosclerotic lesions to close dehiscences in the facial canal in the oval window area [13, 21].

Apart from the analysis of coincidence between facial canal dehiscences and ear diseases, the location, shape, and dimensions of the dehiscences have been the subject of numerous studies. Perez et al. found that 60 % of dehiscences were located in the vicinity of the oval window, mostly in the infero-lateral wall of the tympanic segment of the facial canal [15]. Marquet described the dehiscences of the tympanic segment facial canal (12 %), which were located above the oval window, while the others were found within the antero-inferior side of the second genu [10]. In turn, Takahashi and Sando reported that the majority of dehiscences were located within the posterior half of the oval window (57.0 % of all encountered locations of facial canal dehiscences) on the inferior to infero-medial aspect of tympanic segment [19].

In our material, the dehiscences of the tympanic segment of the facial canal were located mainly in the inferior wall (66.7 % both in infants and adults), rarely in the infero-medial wall (in adults 33.3 %; in infants 16.7 %) and infero-lateral wall (16.7 %, appeared only in infants). All observed dehiscences occupied part of the wall of the tympanic segment which was located above the oval window or slightly backward.

Both Baxter and Kim et al. reported similar percentages of dehiscences within the tympanic segment which were located near the oval window, respectively, 83 and 84.6 % [1, 7]. According to Kim et al., in patients with chronic otitis media in 69.2 % of cases, the lateral wall of the tympanic segment was dehiscent at the oval window area [7]. Similar findings indicating the predominance of the lateral position of the dehiscence were presented by Măru et al. [11].

The previous morphometrical studies revealed that the most common shape of the facial canal dehiscences was elliptic and their length can reach 3 mm. The range of length of the facial canal dehiscences was basically assessed in histological studies [1, 11, 19]. According to Baxter, the widest point of dehiscences within the tympanic segment varied from 0.4 to 3.08 mm [1]. Takahashi and Sando applied light microscopy and computer-aided three-dimensional reconstruction to estimate the parameters of all encountered dehiscences within the facial canal. They found out that the length of the dehiscences ranged from 0.4 to 2.64 mm, width from 0.12 to 1.59 mm, and surface area from 0.03 to 1.87 mm^2^ [19, 20]. In turn, Măru et al. dissected human temporal bones from heads of cadavers and using endoscopic views, and reported that the length of the dehiscences varies from 0.4 to 2.8 mm [11].

In the literature, there is only general information on different shapes of the facial canal dehiscences, however, without determining their percentage. The results of our study confirm the observations of other researchers on the shape and size of dehiscences. We found out that the most frequent shape of the dehiscence (elliptic) occurred in 66.7 % of infants and in 50.0 % of adults. Hence, we identified fusiform- and trapezoidal-shaped dehiscences in infant samples (see Fig. 4c, d) which were not described before in the literature.

Measurements of dehiscences in the tympanic segment which we carried out using micro-CT data revealed that the most frequent length ranged from 0.5 to 1.4 mm both in infants (50.0 %) and adults (75.0 %). Dehiscences longer than 1.4 mm (1.5–3.5 mm) were more frequent in infants (50.0 %) than in adults (25.0 %). This could be due to the fact that in some infants, the wall of the tympanic segment of the facial canal had not been yet completed [23].

The larger size of the facial canal dehiscences may increase the risks of damage to the exposed facial nerve during surgical treatment within the tympanic cavity. Therefore, preoperative estimation of the facial canal morphology seems to be essential for minimizing negative effects. Nevertheless, in the tympanic segment of the facial canal, some dehiscences may be overlooked in clinical CT scans or volume reconstructions of the middle ear. As it was shown in our study, the most frequent length of the dehiscences in the tympanic segment is less than 1.5 mm, particularly in adults (75.0 %), and such small structures are hardly visible in clinical tomographs, because the resolution capabilities of these instruments are on the order of 1 mm.

## Conclusions

Applied computed microtomography clearly showed the complex course of the facial canal and allowed for precise detection of the dehiscences within the tympanic segment of the facial canal both in infant and adult samples harvested from temporal bones. In the majority of cases, the dehiscence occurred in the inferior wall of the tympanic segment of the facial canal and was elliptic in shape, with their long diameter ranging from 0.5 to 3.5 mm. The presence of dehiscences can increase the risk of spreading pathogens across the facial canal and makes the facial nerve more vulnerable to damage. Data obtained from the micro-CT can help to determine what kind of the dehiscences (their size, shape and location) enhances the risk of the facial nerve destruction. In addition, we suggest that micro-CT study can revise or establish new criteria for defining and classification of the facial canal dehiscences. Clinical evaluation of the dehiscences should consider such cases of the wall of the facial canal which may appear seemingly as dehiscent.
